# Supplementary material for: Effectiveness Evaluation of Repetitive Transcranial Magnetic Stimulation Therapy Combined with Mindfulness-Based Stress Reduction for People with Post-Stroke Depression: A Randomized Controlled Trial
Source: Int J Environ Res Public Health. 2023 Jan 4;20(2):930. doi: 10.3390/ijerph20020930 (PMC9858717; doi:10.3390/ijerph20020930)
Supplement: Supplementary file 1 [file ijerph-20-00930-s001.zip › ijerph-2084640-supplementary.pdf]

Mindfulness-Based stress reduction intervention content

Time	Goal	Content
Pilot course	Introducing team members to one another	introducing the origin of mindfulness, class structure, and requirements, and teaching everyone how to download and utilize Tencent meeting; seven attitudes and precautions for maintaining mindfulness during stroke rehabilitation. Consider the purpose of practicing mindfulness.
first Session	Exploring unique stressors in stroke rehabilitation	Three-minute meditation practice： Close your eyes softly, set a backrest behind your chair or wheelchair, sit comfortably without collapsing, concentrate on your breathing, and feel a rumble in your tummy. The patient assumes a seated position for a 10-minute body scanning exercise, and he is instructed to try to remain awake during the exercise. If he is asleep, he can open his eyes and perform exercises, place a pillow or blanket on the affected limb, and hold a sphere in the affected hand. Combined with meditation practice, the patient scans his or her attention from the foot to the head while breathing and observes the affected side's bodily sensation.
2 nd Session	To develop an awareness of the body, to understand the limitations of the body	Breathing space practice for three minutes: gently close your eyes and focus on the sensations of cool and warm air entering and exiting your nasal cavity. The entire technique does not control breathing and instead helps patients to concentrate on a certain region of the body in order to feel the sensation of breathing in that portion. Exercises for Chair Yoga include gentle stretching of the limbs, hands, feet, and neck. Exercises are either performed for a specific aim or to sense and feel. Patients with inactive limbs are encouraged to visualize their movements. Invite to share a positive stroke recovery experience
3rd Session	Understand stress and its physical and mental effects	stop practice: s - stop what you are doing, t - conscious breathing, o - aware of anything rising within yourself, including any thoughts, emotions, or body sensations, p - prompt you to move forward deliberately; Participants can practice in a safe environment while being followed by their families, feel the sensation of contact between their feet and the ground, and experience the raising, moving, and lowering of their feet as well as the movement of their joints and muscles. Patients who are unable to walk should not sit on a chair too quickly, visualize the entire process of walking, and move the limbs that can be moved. Invitation to discuss additional aspects contributed by a pleasant and a sad occurrence
4th Session	Learning Mindfulness Responses to Stress	15 minutes of body scanning; to discuss with patients the causes of the stress connected with rehabilitation and the behavior of coping with stress as opposed to reaction, to correct negative bias, and to identify typical pitfalls leading to self-defeat in stroke rehabilitation; An invitation to discover new methods to confront difficult emotions and concerns and the task of growing bravery; illness rehabilitation tools, support, and aid that may be sought.
5th Session	Understanding communication patterns, learning to bring mindfulness into relationships	RAIN practice: R-recognition, recognition of inner emotions, A-acceptance, allowing oneself to have such emotions; I-inquiry, in-depth exploration of why such emotions occur; N-non-identity, realizing that negative emotions are our guests, we can send him away friendly; invite experiences that express communication difficulties (the challenge of communicating with caregivers, family, friends, and healthcare providers about your experiences and needs during stroke recovery); in the face of communication difficulties, invite to express, reflect and admit: Who am I? How is my interpersonal relationship affected? What do I need to take care of my emotions？
6th Session	Encourage mindfulness in life	For meditation practice, you may sit, lie down, or stand in any position. Guide you through a meditation about love, the mountains, or a lake. Review and repeat all formal tasks in the course, including the seven mindfulness attitudes.
